# Acetyl salicylic acid augments functional recovery following sciatic nerve crush in mice

**DOI:** 10.1186/1749-7221-2-3

**Published:** 2007-02-04

**Authors:** Prasanna Kumar T Subbanna, C G Prasanna, Bhagawat K Gunale, Manoj G Tyagi

**Affiliations:** 1 Department of Pharmacology & Clinical Pharmacology, Christian Medical College, Vellore-632002, India

## Abstract

Cyclin-dependent kinase 5 (CDK-5) appears to play a significant role in peripheral nerve regeneration as CDK-5 inhibition retards nerve regeneration following nerve crush. Anti-inflammatory drug acetyl salicylic acid elevates CDK-5 and reduces ischemia – reperfusion injury in cultured neurons. In this study we have evaluated the effect of acetyl salicylic acid on functional recovery following sciatic nerve crush in mice. Eighteen Swiss albino mice underwent unilateral sciatic nerve crush. Test animals received acetyl salicylic acid (100 mg/kg/day, n = 6 or 50 mg/kg/day, n = 6) and control animals (n = 6) received normal saline for 14 days following surgery. Functional recovery was assessed with improvement in Sciatic Function Index, nociception and gait. In comparison with normal saline treatment, acetyl salicylic acid (100 mg/kg/day) significantly improved functional recovery following sciatic nerve crush. Anti-inflammatory drug acetyl salicylic acid appears to be a promising agent for treating peripheral nerve injuries and hence elucidation of its neuroprotective pathways is necessary.

## Background

The injured adult mammalian peripheral nerves, in contrast with axons injured inside central nerve tracts, show vigorous regeneration [1]. The exact physiological and molecular signals involved in inducing the regenerative process are largely unknown. In addition to the induction of transcription factors, adhesion molecules, growth associated proteins and structural components required for axonal elongation, intracellular signalling molecules that control cell cycle and differentiation appears to play a major role in nerve regeneration process [1].

Cyclins and the cyclin dependent kinases (CDKs) play a central role in regulating the cell cycle progression in all eukaryotic organisms [2]. Cyclin-dependent kinase – 5 (CDK-5) is a member of these cyclin-dependent kinase family of serine/threonine kinases. CDK-5 along with its activators, p35 and p39, is predominantly expressed in post-mitotic neurons [3]. CDK-5 appears to be involved in active reorganization of the actin cytoskeleton during neurite outgrowth [4]. Enhanced CDK-5 activity and expression of p35 are associated with differentiation of cultured neuronal cells as well as accelerated neurite outgrowth [4]. Namgung et al [5] reported a high expression of CDK-5 and p35 in regenerating nerves. In their experiments inhibition of CDK-5 activity, through CDK-5 inhibitors roscovitine and olomoucine, led to reduction in CDK-5 activity and retardation of nerve regeneration [5].

Non-steroidal anti-inflammatory agent acetyl salicylic acid (ASA), in addition to its well known inhibitory action on cyclooxygenases, affects several cellular signalling pathways involved in regulation of cellular proliferation and differentiation [6]. One of the newly identified actions of ASA is being the induction of p 35 synthesis and activation of CDK-5 [6]. ASA has shown a neuroprotective effect in an *in-vitro *model of neuronal ischemia reperfusion injury [6]. However its effect on peripheral nerve injuries is unknown.

In this study we have evaluated the effect of ASA at two doses (100 mg/kg/day and 50 mg/kg/day) on functional recovery following peripheral nerve injury using mouse sciatic nerve crush model.

The following drugs were used for this study: Urethane (Sigma, USA), Normal Saline (Baxter, India) and ASA (Alta Labs, India).

Swiss albino mice (25 – 30 gms) of both sexes were randomly allocated into three different treatment and control groups. Animals received food and water *ad libitum *and were kept on a 12-hour light/dark cycle. Animals were kept under the accordance of protocols approved by the institutional animal care and use committee.

Mice were subjected to sciatic nerve crush as described earlier [7]. In brief adult mice were anesthetized with 150 mg/100 g intraperitoneal urethane. The area above the right lower thigh was shaved and sterilized with betadine and 70% surgical spirit. A 1 cm incision was made in the skin above the lower thigh between the gluteus maximus muscle and the biceps femoris muscle. The muscles were teased apart with scissors and the sciatic nerve exposed. Sciatic nerve was placed in a 1 mm wide needle holder and crushed for 20 sec. The holder was rotated 90° and the crush was repeated at the same site. The nerve was replaced under the muscle and the incision was sutured. Completeness of the crush was established by examining the loss of sensory and motor function in the operated limb. Digits in the operated limb were pinched using a blunt forceps. Absence of foot withdrawal and vocalization was noted as loss of sensory and motor function. For sham controls the sciatic nerve of the right hind limb was surgically exposed but no crush was made.

The animals in three groups (n = 6 each) received normal saline (0.9 % NaCl, 0.5 ml, ip), ASA (50 mg/kg/day, ip) and ASA (100 mg/kg/day, ip) respectively for 14 days following surgery.

Evaluation of sciatic function index (SFI) [8] and gait [7] was done on day 0 i.e. before surgery and on days 1 and15 following surgery. Mice were held by the chest and their hind feet were pressed down onto a stamp pad soaked with water soluble black ink. Mice were immediately allowed to walk along a confined walkway 6 cm wide by 30 cm long with a dark shelter at the end of the corridor leaving its foot prints on the paper that is cut to the appropriate dimensions and placed on the floor of the corridor. The tracks were evaluated for three different parameters: (1) distance from the heel to the third toe, the print length (PL); (2) distance from the first to the fifth toe, the toe spread (TS); and (3) distance from the second to the fourth toe, the intermediary toe spread (ITS). All three measurements were taken from the experimental (E, undergoing sciatic nerve crush) and normal (N) limbs. Using the following formula derived by Bain et al [9] SFI was calculated as,

SFI = -38.3 [(EPL - NPL)/NPL] + 109.5 [(ETS - NTS)/NTS] + 13.3 [(EIT - NIT)/NIT] - 8.8

The SFI was analysed as: An SFI equal to 100 indicates significant impairment, whereas an SFI oscillating around 0 is considered to reflect normal function.

Animals were allowed to walk on a platform as well as on an inclined plane for 2 min each. Subjective scores were assigned on the basis of hind limb movement and its posture while ambulating. Mice moving both the hind limbs uniformly given 3, if the operated limb was moving with deformity it received 2, scored as 1 if the operated limb was moving seldom and 0 – when no movement was seen in the operated hind limb.

Nociceptive function was evaluated by observing the withdrawal reflex of the hind limb and vocalization in response to noxious stimulation like mechanical stimulation (pinch test) and pricking the plantar aspect of the lateral part of the foot with a needle [7]. Animals were evaluated daily, till the recovery of nociceptive function.

The entire regimen was repeated twice and then all the values from multiple experiments were averaged. Statistical evaluation was conducted using multiple comparisons and Mann Whitney U test. Data are depicted as mean ± sd. 'P' values < 0.05 were considered significant.

In all animals SFI score prior to surgery was 0, gait score was three and nociceptive function was intact. Following sciatic nerve crush in all animals, except sham controls, SFI scores became 100 and gait scores were reduced to 0 and there was loss of nociceptive function. In sham controls there was no change in SFI scores and gait scores, while nociceptive function remained intact.

There was a spontaneous recovery of sensorimotor function in normal saline treated mice as shown by reduction in SFI scores, improvement in gait scores and recovery of nociceptive function (table 1). There was no significant difference, in the functional parameters, between animals treated with 50 mg/kg/day of ASA and animals receiving normal saline treatment. However animals treated with 100 mg/kg/day of ASA showed statistically significant reduction in SFI scores and improvement in gait and exhibited an early recovery in nociceptive function (table 1).

**Table 1:** Improvement in sensorimotor function following sciatic nerve crush

**Treatment groups **(n = 6 animals in each group)	**Improvement in SFI scores **(in %, as on day 15)	**Improvement in gait scores **(in %, as on day 15)	**Time taken for sensory recovery **(number of days)
Normal saline	38.2 ± 1.8	25.12 +/- 4.8	17.80 +/- 3.7
Acetyl salicylic acid (50 mg/kg/day)	41.7 ± 2.4	31.3 ± 1.7	16.1 ± 3.1
Acetyl salicylic acid (100 mg/kg/day)	55.3 ± 1.7*	48.35 +/-1.7*	14.05 +/- 2.0*

Data are depicted as mean ± SD. '*P*' values: < 0.05; * vs control (Mann-Whitney U test with multiple comparisons)

ASA is one of the most widely used analgesic, anti-pyretic and anti-inflammatory drug. ASA exerts these effects through inhibition of cyclooxygenases (COX) [6]. However novel COX-independent actions of ASA like, inhibition of excitatory amino acid release, NF-kappa beta (Nfkb) translocation to the nucleus and expression of inducible nitric acid synthase (iNOS) following cerebral ischemia are projecting ASA as a promising neuroprotective agent for treating stroke [10].

Our results show that ASA, at anti-inflammatory dose, significantly accelerates functional recovery following peripheral nerve crush. Even though ASA at 50 mg/kg/day dose showed marginally higher functional recovery it was not significant in comparison with normal saline treatment. Hence the neuroprotective action of ASA following peripheral nerve injuries appears to be dose-dependent with maximum benefit seen with 100 mg/kg/day.

Even though our preliminary study shows the neuroprotective action of ASA in peripheral nerve injuries, data regarding the molecular mechanisms leading to the neuroprotective action is still lacking. We have not given the histological and molecular evidence for neuroprotective action of ASA. This may be considered as a limitation to our study. Based on the previous reports describing the role of CDK-5 in nerve regeneration [5] and effect of ASA on CDK-5 [6] it may be assumed that ASA promotes nerve regeneration following peripheral nerve injury through activation of CDK-5. However ASA also affects prostaglandin synthesis, iNOS expression, Nfkb translocation, mitogen activated protein (MAP) kinase pathway etc which can modulate nerve regeneration following peripheral nerve injury. Hence understanding the molecular pathways leading to the neuroprotective action of ASA is necessary.

In conclusion our preliminary study shows that acetyl salicylic acid accelerates functional recovery following peripheral nerve injury and it appears to be a promising agent for treating peripheral nerve injuries. Further studies aimed at understanding the molecular mechanisms involved in the neuroprotective action of acetyl salicylic acid are required.

## Abbreviations

1. CDK – Cyclin dependent kinases

2. ASA – Acetyl salicylic acid

3. SFI – Sciatic function index

4. PL – Print length

5. TS – Toe spread

6. IT – Intermediary toe spread

7. COX – Cyclooxygenase

8. NFkB – NF-kappa beta

9. iNOS – Inducible nitric oxide synthase

10. MAP kinase – Mitogen activated protein kinase

## Competing interests

The author(s) declare that they have no competing interests.

## Authors' contributions

1. PKTS – Concept, Experiments, Data analysis, Manuscript preparation

2. PCG – Concept, Experiments, Data analysis

3. BKG – Concept, Experiments, Data analysis

4. MGT – Concept, Data analysis, Manuscript preparation
